# Ccl5 Mediates Proper Wiring of Feedforward and Lateral Inhibition Pathways in the Inner Retina

**DOI:** 10.3389/fnins.2018.00702

**Published:** 2018-10-12

**Authors:** D’Anne S. Duncan, Rebecca L. Weiner, Carl Weitlauf, Michael L. Risner, Abigail L. Roux, Emily R. Sanford, Cathryn R. Formichella, Rebecca M. Sappington

**Affiliations:** ^1^Vanderbilt Eye Institute, Vanderbilt University Medical Center, Nashville, TN, United States; ^2^Department of Pharmacology, Vanderbilt University School of Medicine, Nashville, TN, United States; ^3^Department of Ophthalmology and Visual Sciences, Vanderbilt University School of Medicine, Nashville, TN, United States

**Keywords:** Ccl5, chemokine, retinal ganglion cell, amacrine cell, bipolar cell, gustducin, PKCα

## Abstract

The β-chemokine Ccl5 and its receptors are constitutively expressed in neurons of the murine inner retina. Here, we examined the functional and structural significance of this constitutive Ccl5 signaling on retinal development. We compared outcomes of electrophysiology, ocular imaging and retinal morphology in wild-type mice (WT) and mice with Ccl5 deficiency (*Ccl5^-/-^*). Assessment of retinal structure by ocular coherence tomography and histology revealed slight thinning of the inner plexiform layer (IPL) and inner nuclear layer (INL) in *Ccl5^-/-^* mice, compared to WT (*p* < 0.01). Assessment of postnatal timepoints important for development of the INL (P7 and P10) revealed Ccl5-dependent alterations in the pattern and timing of apoptotic pruning. Morphological analyses of major inner retinal cell types in WT, *Ccl5^-/-^*, gustducin^gfp^ and gustducin^gfp^/*Ccl5^-/-^* mice revealed Ccl5-dependent reduction in GNAT3 expression in rod bipolar cells as well as a displacement of their terminals from the IPL into the GCL. RGC dendritic organization and amacrine cell morphology in the IPL was similarly disorganized in *Ccl5^-/-^* mice. Examination of the intrinsic electrophysiological properties of RGCs revealed higher spontaneous activity in *Ccl5^-/-^* mice that was characterized by higher spiking frequency and a more depolarized resting potential. This hyperactive phenotype could be negated by current clamp and correlated with both membrane resistance and soma area. Overall, our findings identify Ccl5 signaling as a mediator of inner retinal circuitry during development of the murine retina. The apparent role of Ccl5 in retinal development further supports chemokines as trophic modulators of CNS development and function that extends far beyond the inflammatory contexts in which they were first characterized.

## Introduction

Developmentally, the laminar structure of the retina is formed by timed waves of retinal progenitor cell production and migration that is mediated by numerous transcription factors driving early- and late-born cell types (Ohsawa and Kageyama, 2008; Agathocleous and Harris, 2009; Mattar and Cayouette, 2015). Subsequent induction of additional transcription factors mediates differentiation into major cell type classifications, including photoreceptors, horizontal cells, bipolar cells, amacrine cells, and ganglion cells (Jeon et al., 1998; Ohsawa and Kageyama, 2008; Agathocleous and Harris, 2009). Further differentiation into subtypes of these cells is mediated by induction of additional factors (Reese and Keeley, 2016). These subtypes are defined by differences in expression of neurotransmitters, neurotransmitter receptors, and ion channel classes, i.e., metabotropic versus ionotropic channels (Brandstätter et al., 1998; Huang et al., 2003; Ghosh et al., 2004). It is the proper wiring between subtypes of these broad cell classifications that ensures proper encoding to photon stimuli in the retina from a graded glutamate potential to action potentials that can be interpreted by higher visual centers in the brain.

Retinal ganglion cells, whose axons form the optic nerve, transform the graded glutamate potential into action potentials. The excitatory center of the RGC receptive field is established by glutamatergic input to RGC dendrites from bipolar cells in the IPL of the retina (Ghosh et al., 2004). The physiological properties of the RGC receptive fields are further fine-tuned by inhibitory inputs from amacrine cells that establish the surround of the RGC receptive field (Ghosh et al., 2004). Thus, proper formation of this synaptic triad between excitatory feedforward and lateral inhibitory pathways in the IPL is critical for establishment of the RGC receptive field and subsequent neurotransmission of all visual sensory information. While intrinsic factors mediating development and maturation of bipolar cells, amacrine cells and RGCs are well-documented, extrinsic factors that direct formation of circuits at this critical synapse are largely unknown (Morgan and Wong, 2005).

Our previous work indicated that the β-chemokine Ccl5 and its high-affinity receptors are constitutively expressed by neurons of the inner retina, including RGCs and amacrine cells (Duncan et al., 2017). Like other chemokines, Ccl5, formerly known as RANTES, is classically associated with chemotaxis and immune cell migration (Rossi and Zlotnik, 2000; Appay and Rowland-Jones, 2001). However, studies elsewhere in the CNS suggest that Ccl5 and its receptors are also constitutively expressed by neurons and glia (He et al., 1997; Babcock et al., 2003; Pham et al., 2005; Subileau et al., 2009; Sorce et al., 2011; Fe Lanfranco et al., 2018) and may play a role in neuron survival, development and neurotransmission (Galasso et al., 1998; Mennicken et al., 2002; Gamo et al., 2008; Sorce et al., 2010, 2011; Tripathy et al., 2010).

Here, we report the surprising finding that the β-chemokine Ccl5 mediates critical interactions between bipolar cells, amacrine cells and RGC dendrites that are necessary for appropriate development and wiring of the bipolar cell-amacrine cell-RGC circuit. We found that Ccl5 deficiency in *Ccl5^-/-^* mice results in thinning of the inner retina, particularly the INL and IPL. Ccl5 deficiency altered the pattern and timing of cell migration and apoptotic pruning during late-stage development. Assessment of major cell classifications in the mature inner retina revealed Ccl5-dependent changes in: (1) rod bipolar cell phenotypes, including displacement of their terminals from the IPL into the GCL, (2) RGC dendritic organization, and (3) amacrine cell morphology in the IPL. Examination of the intrinsic electrophysiological properties of RGCs revealed higher spontaneous activity in *Ccl5^-/-^* mice that was characterized by higher spiking frequency and a more depolarized resting potential. This hyperactive phenotype could be negated by current clamp and correlated with both membrane resistance and soma area. Overall, our findings identify Ccl5 signaling as a mediator of inner retinal circuitry during development of the murine retina. The apparent role of Ccl5 in retinal development further supports chemokines as trophic modulators of CNS development and function that extends far beyond the inflammatory contexts in which they were first characterized.

## Materials and Methods

### Animals

This study was conducted in accordance with regulations set forth in the ARVO Statement for the Use of Animals in Ophthalmic and Vision Research. Animal protocols were approved by the Institutional Animal Care and Use Committees of the Vanderbilt University Medical Center. Breeding pairs of C57BL/6 and *Ccl5^-/-^* mice were obtained from Charles River Laboratories (Wilmington, MA, United States) and Jackson Laboratories (Bar Harbor, ME, United States), respectively. Breeder pairs of Gusducin reporter mice (Gus^gfp^; Huang et al., 2003) were obtained as a generous gift from Dr. Benjamin Reese (University of Southern California, Santa Barbara, CA, United States). In the Gus^gpf^ mouse, GFP is expressed under the promoter for GNAT3, which is expressed by Type 7 cone bipolar cells and rod bipolar cells (Huang et al., 2003). Mouse colonies were established and maintained in-house at Vanderbilt University Medical Center. We crossed Gus^gfp^ mice with *Ccl5^-/-^* mice to establish and maintain a colony of Gus^gfp^/*Ccl5^-/-^*. All colonies were maintained on a 12 h light, 12 h dark cycle with food and water available *ad libitum*. We conducted experiments in adult mice at 2–4 months of age. For developmental series, samples were obtained from pups at postnatal days 7 and 10.

### Tissue Procurement and Preparation

For fresh tissue, mice were sacrificed by cervical dislocation followed by decapitation. For protein and gene analyses, whole eyes were enucleated, flash-frozen and stored at -80°C until use. For electrophysiological recordings, eyes were enucleated and retina dissected for immediate use. For fixed tissue, mice received an overdose of pentobarbital (200 mg/kg; Hospira, Inc., Lake Forest, IL, United States) and were sacrificed by *trans*-cardial perfusion with 1X phosphate buffered saline (PBS; Fisher Scientific; Pittsburg, PA, United States) followed by 4% paraformaldehyde (PFA; Electron Microscopy Sciences, Hatfield, PA, United States). Whole eyes were enucleated and optic nerve and brain harvested. Whole eyes and brain were post-fixed for 1 h in PFA. Optic nerves were post-fixed at least 48 h in 2.5% glutaraldehyde. For paraffin retina sections, 6 μm serial sections of the entire globe were obtained. For cryosections, whole eyes were cryoprotected in a sucrose series, frozen and sectioned on a cryostat at 10 or 40 μm thickness. To obtain serial sections of SC, cortices were removed and brains were cryoprotected in a sucrose series, frozen, and sectioned (50 μm) on a sliding microtome. For semi-thin sections of optic nerve, distal and proximal segments of optic nerve were embedded in epon and sectioned at 700 nm thickness on an ultra-microtome.

### Retinal Morphology and Layer Thickness

To visualize longitudinal, cross-sections of the retina *in vivo*, we utilized spectral domain ocular coherence tomography (SD-OCT), as previously described (Echevarria et al., 2017). Briefly, mice were anesthetized with an intraperitoneal injection of ketamine and xylazine, and pupils were dilated using 0.5% Tropicamide (Akorn, Inc., Lake Forest, IL, United States) and kept moist using Systane Ultra. B-line scans of retinas were imaged at the level of the OD (eccentricity = 0) using the Bioptigen ultra-high resolution spectral domain OCT system, Envisu class R2200 and a mouse retina bore (Bioptigen, Morrisville, NC, United States). Representative images of C57BL/6 (*n* = 5) and *Ccl5^-/-^* (*n* = 5) retinas were collected, and layer-specific measurements of the retinal nerve fiber layer (RNFL), IPL, INL, OPL, and ONL were analyzed using In VivoVue Diver 2.0 software (Bioptigen). Briefly, a 5 × 5 quadrant overlaid each representative image to measure approximately 25 positions of the retina. Each layer was manually identified at each position, and specific layer thickness, measured in μm, was averaged for each image by experimenters blind to genotype.

### Quantification of Developmental Apoptosis

We performed TUNEL labeling in whole eye cryosections from C57Bl/6 and *Ccl5^-/-^* mice at postnatal days 7 and 10, using a commercially available *in situ* apoptosis detection kit (Cat No. 11684795910; Sigma). TUNEL labeling was performed as per manufacturer’s instructions. Briefly, whole eye sections were permeabilized in 0.1 M Citrate buffer, pH 6.0 by microwave irradiation for 1 min. Slides were rinsed in ddH_2_O followed by PBS. Sections were incubated in blocking serum (3% BSA+ 20% normal bovine serum in 0.1 M Tris-HCl, pH 7.5) for 30 min at room temperature. After PBS washes, sections were incubated for 60 min at 37°C in TUNEL reaction mixture containing terminal deoxynucleotidyl transferase and fluorophore-conjugated nucleotides. Sections were then washed in PBS, counterstained with the nuclear dye DAPI (Life Technologies) and mounted with Fluoromount-G (Southern Biotech; Birmingham, AL, United States). Fluorescent images of retina sections were taken with an inverted epifluorescent microscope (Nikon Instruments) at similar eccentricities (mid-peripheral) across samples. The number of TUNEL+ and DAPI+ cells were counted in their respective retinal layers by at least two independent experimenters blind to genotype, using ImageJ 1.47v. To accurately depict the rate of apoptosis in each layer, TUNEL reactivity is presented as a percentage of the total number of DAPI+ cells. For IPL measurements, where DAPI+ cells were sparsely observed, fields without DAPI+ or TUNEL+ cells were still considered an apoptotic rate of zero and included in the analysis.

### Immunohistochemistry

Immunohistochemistry was performed on whole eye sections of paraffin-embedded or cryostat sections, as previously described (Sims et al., 2012; Echevarria et al., 2013; Duncan et al., 2017). To quench autofluorescence, tissue samples were treated with 0.1% sodium borohydride (Fisher Scientific), blocked in a solution containing 5% normal horse serum (NHS; Life Technologies) and 0.1% Triton X-100 (Fisher Scientific). Tissue was incubated overnight at 4°C in a solution containing the primary antibody (**Table 1**), 3% NHS, and 0.1% Triton X-100 in PBS, followed by a 2-h incubation with the appropriate secondary antibody solution containing 1:200 secondary antibody (donkey anti-mouse, -goat, -rabbit, and -guinea pig; Jackson Immuno, West Grove, PA, United States). Samples were counterstained with DAPI (Life Technologies) and mounted with Fluoromount-G (Southern Biotech; Birmingham, AL, United States). Fluorescent images of retina sections were taken with an inverted epifluorescent microscope (Nikon Instruments) or inverted confocal microscope (Olympus) and analyzed using FluoView Imaging program (Olympus) and ImageJ (NIH). Antibody isotype controls were used to identify non-specific labeling.

**Table 1:** Primary antibodies utilized for immunohistochemistry.

Primary antibody	Concentration	Catalog #	Company
Mouse anti-Brn3a	20 μg/ml	MAB1585	Millipore
Rabbit anti-PKCa	1.67 μg/ml	AB32376	Abcam
Rabbit anti-β-tubulin [neuronal class III β-tubulin (Tuj1) monoclonal]	2.0 μg/ml	802001	BioLegend
Mouse anti-β-tubulin [neuronal class III β-tubulin (Tuj1) monoclonal]	1.33 μg/ml	801201	BioLegend
Mouse anti-syntaxin-1a	100 μg/ml	S0664	Sigma-Aldrich
Goat anti-GFP	1.29 μg/ml	NB100-1770	Novus

### Quantification of Neuronal Density and Layer-Specific Immunolabeling

The total number of bipolar cells (PKCa^+^, Gus^gfp+^), amacrine (syntaxin^+^) and RGCs (Brna3^+^) in C57BL/6, Gus^gfp^, *Ccl5^-/-^* and/or Gus^gfp^/*Ccl5^-/-^* retinas was quantified in 40× or 60× micrographs of immunohistochemically labeled paraffin or cryostat sections, using the ‘freehand selections’ tool in ImageJ 1.47v. The number of immunolabeling+ cells were counted in their respective retinal layers by at least two independent experimenters blind to genotype, using ImageJ 1.47v.

### Anterograde Transport

Anterograde transport to SC of C57BL/6 and *Ccl5^-/-^* mice was performed, as previously described (Crish et al., 2010; Bond et al., 2016; Echevarria et al., 2017). Briefly, mice were anesthetized using 2.5% isoflurane for intravitreal injection of 1 μL of CTB conjugated to Alexa Fluor-594 (1 μL of 1% CTB in sterile PBS solution; Invitrogen). Forty-eight hours post-injection, mice were sacrificed, brain harvested and serial coronal brain sections obtained, as described above. CTB signal in serial coronal brain sections was imaged *en montage* at 10× magnification on an inverted epifluorescent microscope (Nikon Instruments). CTB fluorescent density in the SC was analyzed using ImagePro (Media Cybernetics; Silver Spring, MD, United States). Background intensity for each brain section was set independently for normalization, using pixel strength of the non-retinorecipient SC (layers IV–VII) and the periaqueductal gray. To determine CTB density, area of pixels with CTB signal above background was divided by total pixel area. To further quantify CTB density, colorimetric representation was generated that corresponded with 0% saturation (blue) to 100% saturation (red) at each mediolateral location in the SC section. Using section thickness and intersection distance, we adjoined sections to construct a colorimetric representation of CTB density across the retinotopic SC map. For each SC, we determined the percentage of intact retinotopic map, defined as the percent area with CTB signal ≥70% density.

### Optic Nerve Axon Counts

Axon density and total nerve area was quantified, as previously described (Crish et al., 2010; Sappington et al., 2010; Echevarria et al., 2017). Semi-thin, cross-sections of optic nerve from C57Bl/6 and *Ccl5^-/-^* mice were counterstained with 1% p-Phenylenediamine (PPD) and 1% toluidine blue and imaged *en montage* at 100× magnification on an upright microscope (Olympus). To calculate axon density, a 50 μm × 50 μm grid mask was placed on the montaged image, using NIS elements AR software. The number of axons was manually counted by a blind-observer in 8–10 squares of the grid (Image Pro; Media Cybernetics). Each square counted was equal in area. To measure nerve area, the circumference of the entire nerve was traced in montaged images of optic nerve cross-sections. Nerve area was calculated as the area (mm^2^) within this outline, using NIS elements software. Total number of axons was estimated by multiplying axon counts by total nerve area.

### Whole-Cell Patch-Clamp Recordings

Whole-cell patch-clamp recordings were conducted, as previously described (Weitlauf et al., 2015). Retinas from 6- to 8-week-old C57Bl/6 (*n* = 13) and *Ccl5^-/-^* (*n* = 10) mice were harvested, as described above, hemisected, and stored in oxygenated Ames’ solution (Sigma-Aldrich, St. Louis, MO, United States) at room temperature in the dark for 30 min. Retinal halves were transferred to a temperature-controlled perfusion chamber on an Olympus BX50 upright microscope equipped for fluorescent imaging with a 40× water immersion DIC objective (LUMPlanFI/IR; Olympus, Center Valley, PA, United States). Retinas were submerged with the GCL up and perfused with Ames’ solution at a rate of 2 ml/min in the dark at 24–25°C. Cells in the retinal ganglion layer at mid-peripheral to peripheral eccentricities were whole-cell patch-clamped with borosilicate glass electrodes (5–10 MΩ) containing the following: 130 mM K-gluconate (Sigma), 10 mM KCl, 10 mM HEPES (Sigma), 2 mM MgCl_2_ (Sigma), 1 mM EGTA (Santa Cruz, Dallas, TX, United States), 2 mM Na_2_ATP (Sigma), and 0.3 mM NaGTP (Sigma). We included 1% Lucifer yellow dye (Life Technologies, Carlsbad, CA, United States) in the internal solution to verify that patched cells had an intact axon coursing toward the ONH and to visualize dendritic morphology. We recorded spontaneous action potentials under current-clamp using an AxoClamp 2B microelectrode amplifier outfitted with pCLAMP software (Molecular Devices, Sunnyvale, CA, United States) for a minimum of 4 min. We defined induced cells for cells that did not show a spontaneous firing rate of 0.5 Hz, a 1 s depolarizing step current ranging from 25 to 200 pA was delivered at 0.1 Hz until the firing rate exceeded 3 Hz, for a minimum of 4 min. RGCs that did not achieve a consistent firing rate for a minimum of 4 min were excluded. We used a hyperpolarizing step of 25–200 pA current to calculate membrane resistance. Recordings in which the membrane resistance changed by 20% were not included in the final analysis. For RGC subtype analysis, classifications of ON or OFF or ON/OFF were determined by dendritic stratification. Only RGCs for which stratification in specific sub-laminae of the IPL was readily apparent were included in these analyses.

### Statistical Analysis

All statistical tests were performed with SigmaPlot (Systat Software Inc., San Jose, CA, United States). For RGC electrophysiology analyses, we compared C57BL/6 to *Ccl5^-/-^* mice, using one-way analysis of variance and pair-wise multiple comparisons by Bonferroni *t*-test following normality test validation. For comparisons between RGC electrophysiological properties within genotype, we performed Pearson Product Moment Correlation and linear regression analysis. For all other comparisons, we compared groups using Student’s *t*-test following Shapiro–Wilk normality testing and Brown–Forsythe equal variance analysis. If equal variance testing resulted in *p* < 0.05, groups were compared by Mann–Whitney Rank Sum Test. For all analyses, *p* < 0.05 was considered statistically significant.

## Results

### Ccl5 Deficiency Alters Inner Retina Morphology

**FIGURE 1:**
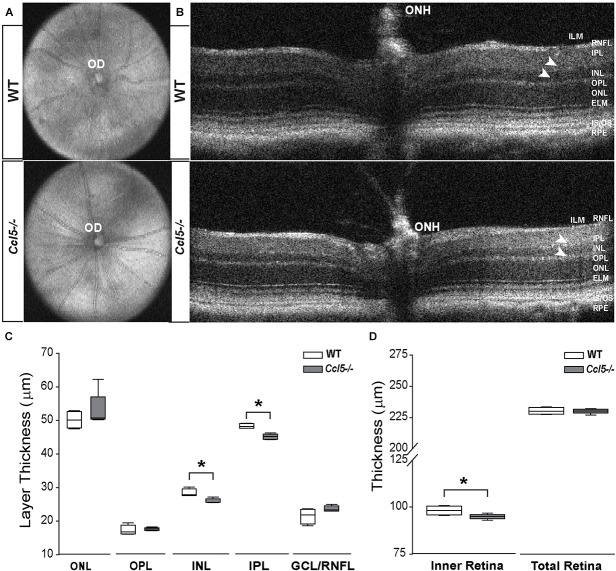
Ccl5 deficiency results in thinning of the inner retina, particularly the INL and IPL. **(A,B)** Representative fundus scans **(A)** and longitudinal B-scans **(B)** of mature (4mo) WT (top) and *Ccl5^-/-^* (bottom) obtained by SD-OCT. **(A)** Fundus scans reveal an intact OD and patterning of vasculature in C*cl5^-/-^* retina, as compared to WT. **(B)** B-scans reveal the presence of all retinal layers from the inner limiting membrane (ILM) to the retinal pigment epithelial (RPE) in *Ccl5^-/-^* mice. However, the INL and IPL appears thinner compared to C57BL/6 retinas (arrowheads). **(C,D)** Box plots of thickness (μm; *y*-axis) for individual layers of the retina (**C**; *x*-axis: ONL, OPL, INL, IPL, and GCL/NFL), the inner retina (**D**; *x*-axis; left) and the total retina (**D**; *x*-axis; right) in WT (white) and *Ccl5^-/-^* (gray) mice. Asterisks indicate *p* < 0.05.

To assess the function of constitutive Ccl5 signaling in retina, we first assessed gross retinal structure *in vivo*, using SD-OCT (Echevarria et al., 2017). In WT and C*cl5^-/-^* mice, fundus images were obtained to examine OD morphology and vascular patterning. B-line scans were obtained to assess gross morphology of the ONH and retinal lamination as well as to measure thickness of individual retinal layers. Fundus images from *Ccl5^-/-^* mice revealed no gross abnormalities in OD morphology or vascular patterning, as compared to WT (**Figure 1A**). Similarly, B-line scans obtained from *Ccl5^-/-^* mice demonstrated no gross abnormalities in ONH morphology or retinal lamination, with all layers of retina apparent and morphologically intact (**Figure 1B**). Quantification of B-line scans revealed slight, but statistically significant, thinning of the INL (26.00 ± 0.69 μm vs. 28.37 ± 1.18 μm) and IPL (45.19 ± 0.84 μm vs. 48.33 ± 0.71 μm) in *Ccl5^-/-^* mice, as compared to WT mice (*p* < 0.05 for both; *n* = 5/group; **Figure 1C**). This resulted in a 4% reduction in inner retina thickness in *Ccl5^-/-^* mice (*p* < 0.05; *n* = 5/group; **Figure 1D**). However, this difference in inner retina thickness did not translate to a change in total retina thickness compared to WT (*p* > 0.05; *n* = 5/group; **Figure 1D**). Together, these data suggest that, while Ccl5 deficiency does not alter the gross laminar structure of the retina, it does alter inner retinal structure, particularly the INL and IPL.

### Ccl5 Deficiency Alters Cell Migration and Apoptotic Pruning During Retinal Development

**FIGURE 2:**
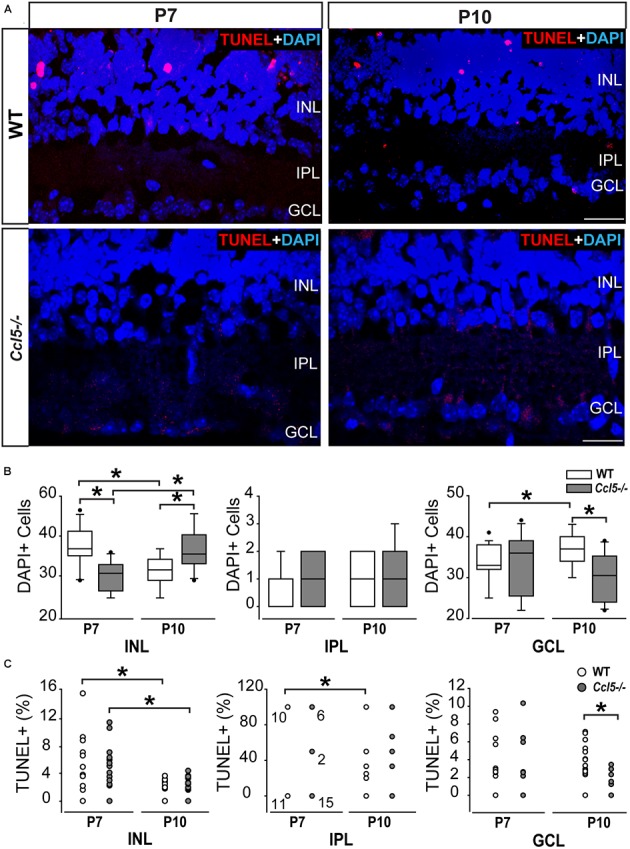
Ccl5 deficiency alters temporal and spatial patterning of inner retinal development. **(A)** Representative micrographs (20×) of TUNEL labeling (red) with DAPI counterstain (blue) in mid-peripheral retina from postnatal day 7 (P7) and 10 (P10) WT and *Ccl5^-/-^* mice. TUNEL reactivity is greater at P7 than at P10 for both genotypes and most apparent in the INL. Scale bar = 20 μm. **(B)** Box plots of the number of DAPI+ cells (*y*-axis) in the INL (*x*-axis; left), IPL (*x*-axis; middle) and GCL (*x*-axis; right) in WT (white) and *Ccl5^-/-^* (gray) retina. Asterisks indicate *p* < 0.05. **(C)** Scatter plots of the percentage of TUNEL+ DAPI+ cells (*y*-axis) in the INL (*x*-axis; left), IPL (*x*-axis; middle) and GCL (*x*-axis; right) of WT (white) and *Ccl5^-/-^* (gray) retina. *N* values are provided for P7 measurements in IPL, which tended to be either 0, 50, or 100% TUNEL+. Asterisks indicate *p* < 0.05.

In the inner retina, the primary neuronal cell types that constitute the INL, IPL, and GCL are bipolar cells, amacrine cells and RGCs, respectively. To determine whether Ccl5-dependent changes in the thickness of inner retina layers arises from altered neurogenesis or apoptotic pruning during retinal development, we quantified the number of DAPI+ cells in inner retinal layers of P7 and P10 WT and *Ccl5^-/-^* pups. At P7, cell genesis and migration of RGCs and amacrine cells is already complete, while bipolar cell genesis and migration is ongoing (Young, 1985; Zhang et al., 2011). By P10, RGC, amacrine cell and bipolar cell genesis and migration are all complete and the cells exhibit adult morphology (Reese, 2011). Quantification of DAPI+ cells indicated that *Ccl5^-/-^* retina contained 21% fewer cells in the INL at P7 than WT retina (*p* < 0.01; *n* = 52–66/group; **Figures 2A,B**). There was no difference in cell number between WT and *Ccl5^-/-^* mice at P7 for the IPL (*n* = 52–66/group) or GCL (*p* > 0.05 for both; *n* = 52–66/group; **Figure 2B**). By P10, the number of cells in the INL decreased by 18% in WT mice, as compared to P7 (*p* < 0.01; *n* = 35–66/group; **Figure 2B**). In contrast, the number of cells in the INL increased by 19% in *Ccl5^-/-^*, as compared to P7 (*p* < 0.01; *n* = 35–66/group; **Figure 2B**). As a result, *Ccl5^-/-^* retina contained 16% more cells in the INL than WT mice at P10 (*p* < 0.01; *n* = 35–66/group; **Figure 2B**). Between P7 and P10, the number of cells in the GCL increased by 9% in WT retina (*p* = 0.01; *n* = 35–66/group; **Figure 2B**). In contrast, there was no change in the number of cells in the GCL of *Ccl5^-/-^* mice between P7 and P10 (*p* > 0.05; *n* = 35–66/group; **Figure 2B**). This resulted in 17% more cells in WT GCL than *Ccl5^-/-^* GCL at P10 (*p* < 0.05; *n* = 35–44/group; **Figure 2B**). In the IPL, we detected a sparse number of DAPI+ cells, which presumably are actively migrating bipolar cells. The number of cells increased by 89% between P7 and P10 in WT retina (*p* < 0.05; *n* = 35–66/group; **Figure 2B**). In contrast, there was no change in the number of cells in the IPL of *Ccl5^-/-^* retina between P7 and P10 (*p* > 0.05; *n* = 35–66/group; **Figure 2B**). Despite changes in cell number in IPL noted in WT retina, there was no difference in the number of DAPI+ cells in the IPL between WT and *Ccl5^-/-^* at P10 (*p* > 0.05; *n* = 35–44/group; **Figure 2B**). These data suggest that changes in mature inner retinal structure is preceded by altered cell density in individual retinal layers, which could arise from either changes in neurogenesis or cell migration.

To determine whether Ccl5-dependent changes in cell numbers within inner retinal layers during the critical developmental period between P7 and P10 arises from altered apoptotic pruning, we performed TUNEL labeling and quantified TUNEL reactivity as % of DAPI+ cells. Qualitatively, TUNEL reactivity was most notable at the P7 timepoint in WT and *Ccl5^-/-^* mice (**Figure 2A**). At P7, we found no difference in the percentage of TUNEL+ cells between WT and *Ccl5^-/-^* across all inner retina layers (*p* > 0.05 for all; *n* = 52–66/group; **Figure 2C**). In the INL, the percentage of TUNEL+ cells decreased between P7 and P10 by 55% in WT mice (*p* < 0.01) and 71% in *Ccl5^-/-^* mice (*p* < 0.01; *n* = 35–66/group; **Figure 2C**). In the IPL, the percentage of TUNEL+ cells decreased between P7 and P10 by 52% in WT mice (66.67 ± 48.8% vs. 31.57 ± 44.6%; *p* < 0.05; *n* = 35–66/group; **Figure 2C**). In contrast, there was no change in the percentage of TUNEL+ cells in the IPL of *Ccl5^-/-^* retina between P7 and P10 (*p* > 0.05; *n* = 35–66/group; **Figure 2C**). In the GCL, there was no change in the percentage of TUNEL+ cells between P7 and P10 in either WT or *Ccl5^-/-^* retina (*p* > 0.05 for both; *n* = 35–66/group; **Figure 2C**). However, the percentage of TUNEL+ cells was 78% lower in *Ccl5^-/-^* retina versus WT retina at P10 (*p* < 0.01; *n* = 35–44/group; **Figure 2C**). Together, these data suggest that Ccl5 deficiency alters the time course and/or magnitude of apoptotic pruning during a critical period of development for inner retinal neurons.

### Ccl5 Deficiency Induces Abnormal Rod Bipolar Cell Differentiation

**FIGURE 3:**
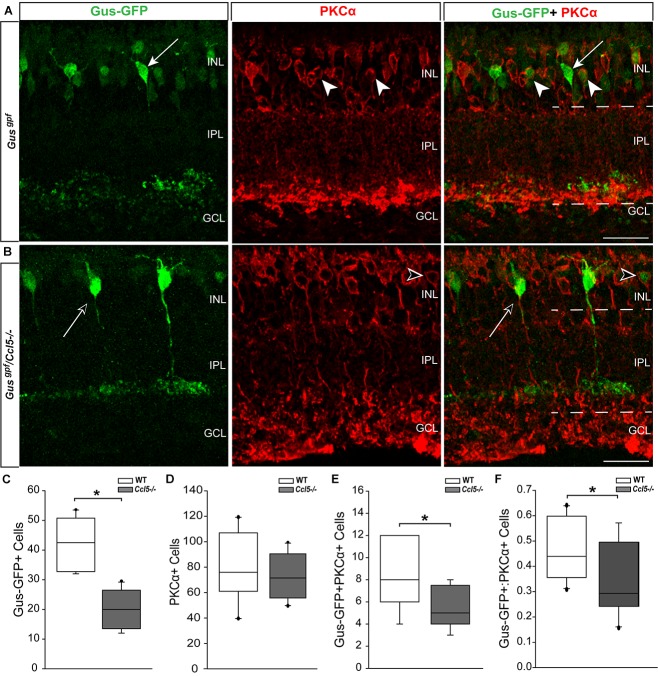
Ccl5 deficiency alters rod bipolar cell phenotypes and morphology. **(A,B)** Representative confocal micrographs (60×) of retina from Gus^gfp^ mice **(A)** and Gus^gpf^/*Ccl5^-/-^* mice **(B)** co-immunolabeled with primary antibodies against GFP (green) and the rod bipolar cell marker PKCα (red). Scale = 20 μm. **(A)** GFP labels PKCα- type 7 cone bipolar cells (filled arrows) and some PKCα+ rod bipolar cells (filled arrowheads) in WT Gus^gfp^ retina. **(B)** In Gus^gpf^/*Ccl5^-/-^*, GFP +/PKCα– cone bipolar cells (unfilled arrow) appear unremarkable. However, GFP+/PKCα+ rod bipolar cells (unfilled arrowhead) appear far less abundant. GFP–/PKCα+ rod bipolar cells appear to terminate in the GCL rather than in the IPL (dotted lines) just below GFP+/PKCα– cone bipolar cells, as seen in WT Gus^gfp^ retina **(A)**. **(C–E)** Box plots depicting the number of Gus-GFP+ **(C)**, PKCα+ **(D)**, and GFP+/PKCα+ **(E)** cells (*y*-axes) in the INL of Gus^gfp^ (white) and Gus^gpf^/*Ccl5^-/-^* (gray) mice. Asterisks indicate *p* < 0.05. **(F)** Box plot depicting the ratio of Gus-GFP+ to PKCα+ bipolar cells (*y*-axis) in WT (white) and *Ccl5^-/-^* (gray) mice. Asterisks indicate *p* < 0.05.

Since cell count and TUNEL analyses suggest that Ccl5 deficiency alters development of the inner retina, we examined inner retina neuron subtypes in mature retina. For bipolar cell analyses, we crossed GUS reporter mice (Gus^gfp^) on a C57Bl/6 background with *Ccl5^-/-^* mice. In GUS-gfp mice, Type 7 cone bipolar cells and a subset of rod bipolar cells express GFP under the gustaducin promoter (Huang et al., 2003). To identify remaining rod bipolar cells, we immunolabled whole eye sections from Gus^gfp^ and Gus^gfp^/*Ccl5^-/-^* mice with antibodies against the rod bipolar cell marker PKCα (**Figures 3A,B**). Qualitatively, GFP+ cone bipolar cells exhibited similar morphology in Gus^gfp^ and Gus^gfp^/*Ccl5^-/-^* mice (arrows; **Figure 3A**). However, GFP+ rod bipolar cells were far less numerous in Gus^gfp^/*Ccl5^-/-^* mice than Gus^gfp^ mice (arrowheads; **Figures 3A,B**). Quantification of the number of Gus-GFP+ cells in the INL of Gus^gfp^ and Gus^gfp^/*Ccl5^-/-^* mice revealed a 53% decrease in the number of Gus-GFP+ bipolar cells in Gus^gfp^/*Ccl5^-/-^* mice, as compared to Gus^gfp^ mice (*p* < 0.01; *n* = 12–19/group; **Figure 3C**). Unlike Gus-GFP+ bipolar cells, PKCα+ rod bipolar cells in Gus^gfp^/*Ccl5^-/-^* mice exhibited altered morphology, where PKCα+ bipolar cells terminated well into the GCL rather than the inner sublamina of the IPL (dotted lines; **Figures 3A,B**). Quantification of the number of PKCα+ bipolar cells revealed that this alteration in bipolar cell terminations was not accompanied by a change in the number of PKCα+ bipolar cells, as compared to WT (*p* > 0.05; *n* = 12–20/group; **Figure 3D**). This suggests that the reduction in Gus-GFP+ rod bipolar cells represents a decrease in GUS expression in rod bipolar rather than a decrease in the actual population of rod bipolar cells. Thus, we quantified the number of Gus-GFP+/PKCα+ rod bipolar cells and the ratio of Gus-GFP+ cells to PKCα+ cells. We found that retina from Gus^gfp^/*Ccl5^-/-^* contained 37% fewer Gus-GFP+/PKCα+ rod bipolar cells than Gus^gfp^ retina (*p* < 0.01; *n* = 12–20/group; **Figure 3E**). This decreased the ratio of Gus-GFP+ cells to PKCα+ cells by 26% (*p* < 0.01; *n* = 12–20/group; **Figure 3F**). Thus, Gus^gfp^/*Ccl5^-/-^* retina contained fewer Gus-GFP+/PKCα+ bipolar cells and more PKCα+ only cells than wild-type Gus^gfp^ retina. Together, these data indicate that Ccl5 deficiency significantly alters the phenotype of mature rod bipolar cells, including reduced expression of GNAT3 and elongation of their terminals beyond the IPL-GCL boundary.

### Ccl5 Deficiency Alters RGC Dendritic Morphology, but Not Density

**FIGURE 4:**
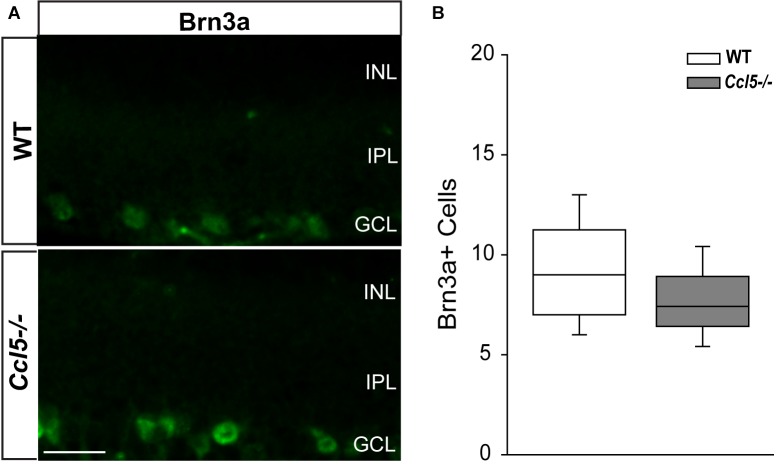
Ccl5 deficiency does not alter RGC density. **(A)** Representative fluoromicrographs of retina from WT (top) and *Ccl5^-/-^* mice (bottom) immunolabeled with primary antibodies against the RGC marker Brn3a reveal similar numbers of Brn3a+ RGCs in both genotypes. Scale = 20 μm. **(B)** Box plot depicting the number of Brn3a+ cells (*y*-axis) in WT (white) and *Ccl5^-/-^* (gray) mice. Asterisks indicate *p* < 0.05.

**FIGURE 5:**
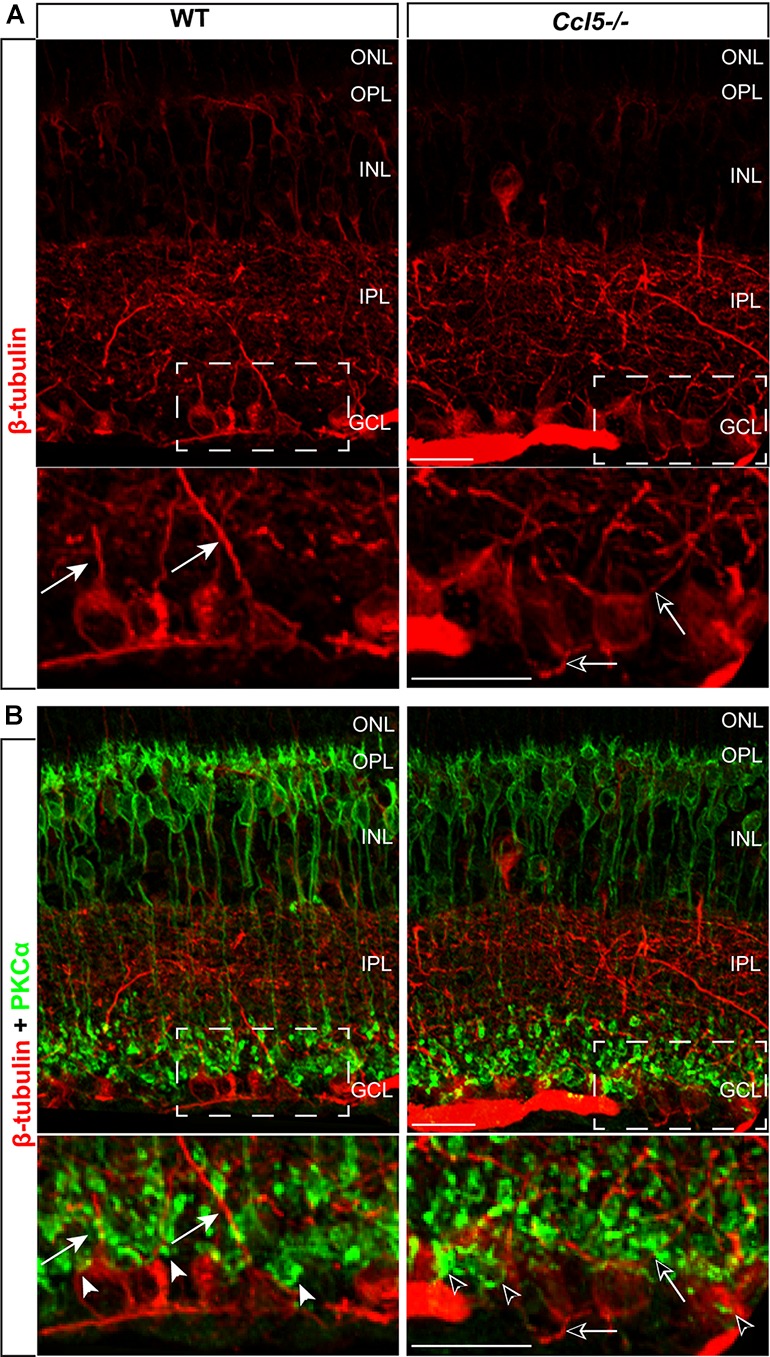
Ccl5 deficiency alters the organizational morphology of RGC dendrites. **(A)** Representative confocal micrographs (60x) of retina from WT (left) and *Ccl5^-/-^* mice (right) immunolabeled with primary antibodies against beta-tubulin (β-tubulin; red) reveal disorganized trajectories of dendritic projections in *Ccl5^-/-^* retina (unfilled arrows), as compared to the uniform, upright trajectory observed in WT retina (filled arrows). Bottom panels are higher magnification views of the outlined area in top panels. Scale = 20 μm. **(B)** Representative confocal micrographs (60×) of retina from WT (left) and *Ccl5^-/-^* mice (right) co-immunolabeled with primary antibodies against β-tubulin (red) and PKCα (green) reveal that terminals of PKCα+ rod bipolar cells (unfilled arrowheads) encroach into the GCL and surround β-tubulin+ RGC soma. In contrast, terminals of PKCα+ rod bipolar cells in WT retina contact only the apical surface of β-tubulin+ RGC soma (filled arrowheads). Bottom panels are higher magnification views of the outlined area in top panels. Scale = 20 μm.

To determine whether Ccl5 deficiency alters RGC maturation, we immunolabeled WT and *Ccl5^-/-^* retina with antibodies against the RGC-specific marker Brn3a and quantified the number of Brn3a+ cells. Brn3a+ RGCs were apparent in the GCL of both WT and *Ccl5^-/-^* retina (**Figure 4A**). Quantification revealed comparable numbers of Brn3a+ RGCs in the GCL in WT and *Ccl5^-/-^* mice (*p* > 0.05; **Figure 4B**). Since Ccl5 deficiency alters the position of rod bipolar cell terminals, we next examined the morphology of RGCs and their dendrites. We immunolabeled WT and *Ccl5^-/-^* retina with antibodies against beta-tubulin III (β-tubulin) and PKCα. RGCs in WT retina exhibit organized, upward trajectories of β-tubulin+ dendrites (filled arrows; **Figure 5A**). In contrast, RGCs in *Ccl5^-/-^* retina exhibit β-tubulin+ dendrites with multi-directional trajectories (unfilled arrows; **Figure 5A**). Co-immunolabeling with PKCα demonstrates clear separation between terminals of PKCα+ rod bipolar cells and β-tubulin+ RGC soma (filled arrowheads; **Figure 5B**). In *Ccl5^-/-^* retina, terminals of PKCα+ rod bipolar cells are interspersed between and around β-tubulin+ RGC soma (unfilled arrowheads; **Figure 5B**). β-Tubulin+ RGC dendrites are closely associated with these terminals, suggesting that aberrant trajectories of RGC dendrites reflect improper positioning of rod bipolar cell terminals (unfilled arrowheads vs. unfilled arrows; **Figure 5B**).

### Ccl5 Deficiency Does Not Alter the RGC Projection

**FIGURE 6:**
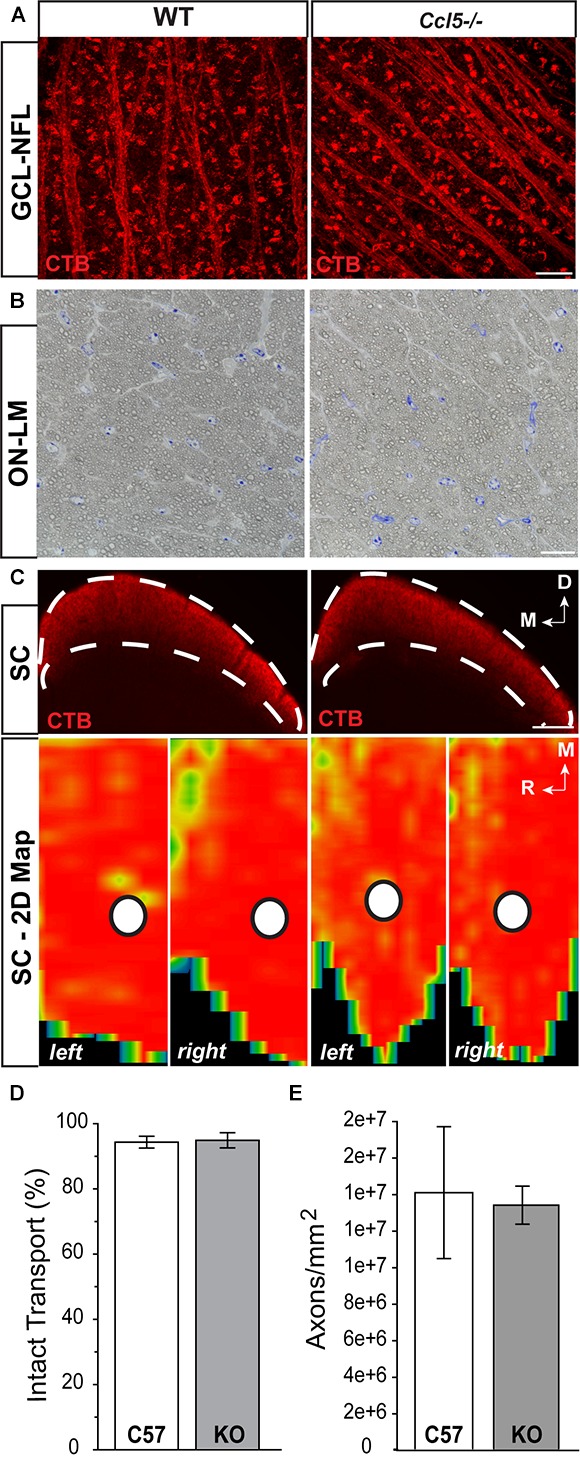
Ccl5 deficiency does not alter the RGC projection to the superior colliculus. **(A)** Representative confocal fluoromicrographs (60×) of CTB neural tracing (red) in the GCL and NFL of whole mount retina from WT (left) and *Ccl5^-/-^* (right) mice. Both WT and *Ccl5^-/-^* retina demonstrate comprehensive uptake and transport of CTB in RGC soma and axons, respectively. Scale = 20 μm. **(B)** Representative micrographs (100×) of optic nerve cross-sections from WT (left) and *Ccl5^-/-^* (right) mice. Morphology of (RGC axons appear unremarkable in *Ccl5^-/-^* optic nerve, as compared to WT optic nerve. Scale = 100 μm. **(C)** Representative coronal sections through the superior colliculus (SC; top) and respective 2-D retinotopic heat maps of CTB labeling in the entire SC (left and right lobes; bottom) in WT (left panels) and *Ccl5^-/-^* (right panels) mice. Top: Dotted lines indicate retinorecipient layers in the superficial SC (top panels). Dorsal (D) and medial (M) orientations are indicated. Scale = 300 μm. Bottom: Density of the CTB signal for heat maps range from 0% (blue) to 50% (green) to 70% (yellow) to 100% (red). White circles indicate the OD representation in 2-D SC maps (bottom panels). Medial (M) and rostral (R) orientations are indicated. **(D)** Bar graph of average percent intact transport (>70% density of CTB signal; red/yellow areas) to the SC in WT (white) and *Ccl5^-/-^* (gray) mice reveals no difference between genotypes (*p* > 0.05). Error bars indicated standard deviation. **(E)** Bar graph of mean axon density in optic nerves from WT (white) and *Ccl5^-/-^* (gray) mice reveals no difference between genotypes (*p* > 0.05). Error bars indicate standard deviation.)

To determine whether Ccl5 deficiency impacts the primary RGC projection, we examined NFL and optic nerve morphology and RGC connectivity with distal brain structures. We performed neural tracing of the RGC projection with fluorophore-conjugated cholera toxin β-subunit (CTB). In rodents, approximately 90% of RGC axons terminate in the SC, with the remaining 10% terminating in the lateral geniculate nucleus, tectum and hypothalamus. Thus, we quantified anterograde transport of CTB from RGC soma to retinorecipient layers of the SC. Comparable to WT, CTB was apparent in RGC soma across the GCL and in the unmyelinated segment of RGC axons in the NFL (**Figure 6A**). Optic nerve integrity appeared intact with comparable axon myelination and glial cell content in semi-thin cross-sections of optic nerve from *Ccl5^-/-^* and WT mice (**Figure 6B**). CTB tracing revealed appropriate anterograde transport along the optic nerve to its termination in the SC in both WT and *Ccl5^-/-^* mice (**Figure 6C**). Two-dimensional reconstruction of CTB labeling in the SC and subsequent quantification of CTB labeling density revealed comparable innervation of retinorecipient layers in the SC between WT and *Ccl5^-/-^* mice (*p* > 0.05; *n* = 8–9/group; **Figures 6C,D**). Quantification of RGC axon density further indicated no difference between WT and *Ccl5^-/-^* mice (*p* > 0.05; *n* = 8–12/group; **Figure 6E**). These data demonstrate that, while Ccl5 deficiency alters the dendritic compartment of RGCs, it does not alter RGC density or their connectivity via the optic projection.

### Ccl5 Deficiency Alters Amacrine Cell Morphology

**FIGURE 7:**
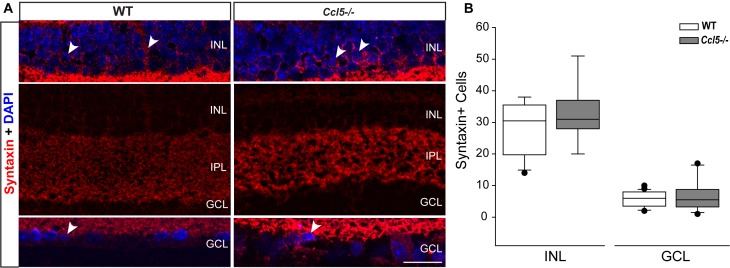
Ccl5 deficiency alters the morphology and organization of amacrine cells. **(A)** Representative confocal micrographs (60×) of syntaxin-1A immunolabeling (red) with DAPI counterstain (blue) in INL (top), IPL (middle) and GCL (bottom) of retina from WT (left) and *Ccl5^-/-^* (right) mice. Syntaxin immunolabeling, particularly in the IPL, appears more condensed in the thinner IPL of *Ccl5^-/-^* retina, as compared to WT retina. Scale = 30 μm. **(B)** Box plot of the number of syntaxin+ cells in the INL (*y*-axis) of WT (white) and *Ccl5^-/-^* (gray) retina indicates no significant difference between genotypes. Error bars indicate standard deviation.

Lateral inhibition in the inner retina is provided by amacrine cells, which are post-synaptic to bipolar cells and pre-synaptic partners to RGCs. To determine whether amacrine cell migration and differentiation is also affected by Ccl5 deficiency, we immunolabeled whole eye sections of WT and *Ccl5^-/-^* mice with antibodies against the amacrine cell marker syntaxin-1a. In WT mice, syntaxin immunolabeling was apparent in amacrine cell soma in the INL and GCL (arrowheads; **Figure 7A**) and, most markedly, in amacrine cell processes in the IPL (**Figure 7A**). In *Ccl5^-/-^* mice, the overall pattern of syntaxin+ labeling was similar to WT (**Figure 7A**). However, syntaxin labeling in the IPL of *Ccl5^-/-^* mice appeared more intense and concentrated, with individual processes of amacrine cells more apparent than in WT mice (**Figure 7A**). Quantification of syntaxin+ amacrine cell soma in the INL and GCL revealed no difference between WT and *Ccl5^-/-^* mice (*p* > 0.05; *n* = 25–27/group; **Figure 7B**). This suggests that disorganization and altered substructure of the IPL in *Ccl5^-/-^* is not due to major alterations in amacrine cell number.

### Ccl5-Deficiency Increases Spontaneous Activity of RGCs

**FIGURE 8:**
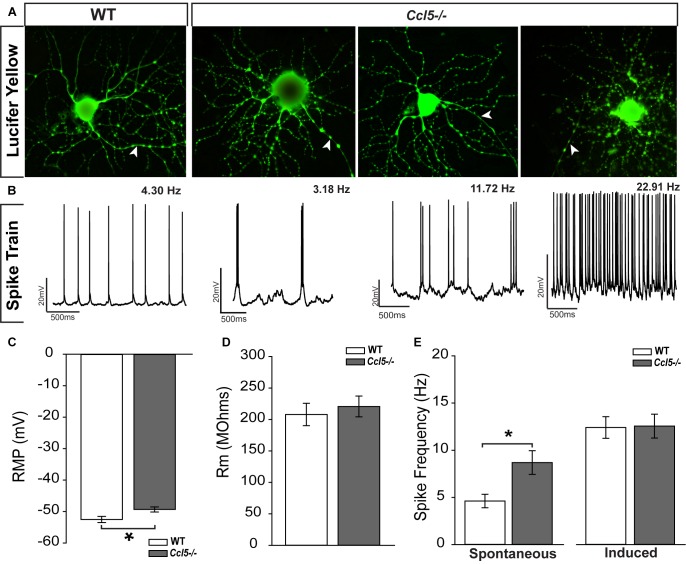
*Ccl5^-/-^* deficiency increases spontaneous activity in RGCs. **(A)** Representative micrographs of RGCs from WT (right) and *Ccl5^-/-^* (left) filled with 1% Lucifer yellow during whole-cell patch-clamp recording reveal the presence of ramified dendritic trees and intact axons (arrowheads) in both genotypes. **(B)** Representative action potential traces from the cells depicted in **(A)** reveal a broad range of spiking frequencies in RGCs from *Ccl5^-/-^* mice that tend toward higher frequencies. **(C)** Graphical representation of mean intrinsic resting membrane potential (RMP; mV; *y*-axis) in RGCs from WT (white) and *Ccl5^-/-^* (gray) retina RGCs. Error bars indicate standard error. Asterisks indicate *p* < 0.05. **(D)** Graphical representation of intrinsic membrane resistance (Rm; MΩ; *y*-axis) in RGCs from WT (white) and *Ccl5^-/-^* (gray) retina reveals no difference between genotypes. Error bars indicate standard error. **(E)** Graphical representation of mean spiking frequency (Hz; *y*-axis) in spontaneous (left; *x*-axis) and current-induced (right; *x*-axis) conditions for RGCs in WT (white) and *Ccl5^-/-^* (gray) retina. Error bars indicate standard error. Asterisks indicate *p* < 0.05.

The ultimate outcome of retinal processing is the RGC action potential. To determine the functional consequences of Ccl5-dependent changes in inner retinal structure, we characterized the physiological properties of individual RGCs in WT and *Ccl5^-/-^* retinas, using whole-cell patch clamp recordings. As suggested by our histological analyses, cell filling with Lucifer yellow during patch-clamp recording revealed the presence of an intact soma, dendritic tree (arrows) and axons (filled arrows) in both WT and *Ccl5^-/-^* retina (**Figure 8A**). RGCs in WT retina exhibited a range of spontaneous firing rates from 1 to 20 Hz (**Figure 8B**). In contrast, RGCs in *Ccl5^-/-^* mice exhibited a broader range of spontaneous spike rates ranging from 1 to 32 Hz (**Figure 8B**). RGCs in *Ccl5^-/-^* mice exhibited a more depolarized resting membrane potential (RMP) than those in WT retina (49 mV versus 53 mV; *p* < 0.05; *n* = 38–43/group; **Figure 8C**). However, there was no difference in mean membrane resistance (Rm) between the two genotypes (*p* > 0.05; *n* = 37–42/group; **Figure 8D**). As noted qualitatively (**Figure 8B**), the mean spontaneous spiking frequency was almost twofold higher in RGCs from *Ccl5^-/-^* mice than in RGCs from WT mice (*p* < 0.05; *n* = 20–33/group; **Figure 8E**). This was independent of ON/OFF or ON and OFF classification (**Supplementary Figure S1**). To determine whether this increased level of spontaneous activity is intrinsic to RGCs or arises from presynaptic circuitry, we applied depolarizing currents until the induced spike frequency exceeded 3 Hz. When the threshold firing rate of 3Hz was met or surpassed, the firing rates of RGCs from WT and *Ccl5^-/-^* retina were indistinguishable (*p* > 0.05; *n* = 14–18/group; **Figure 8E**). These data indicate that Ccl5 deficiency leads to a higher baseline activity in RGCs. However, Ccl5 deficiency does not alter RGC responses to applied current.

**FIGURE 9:**
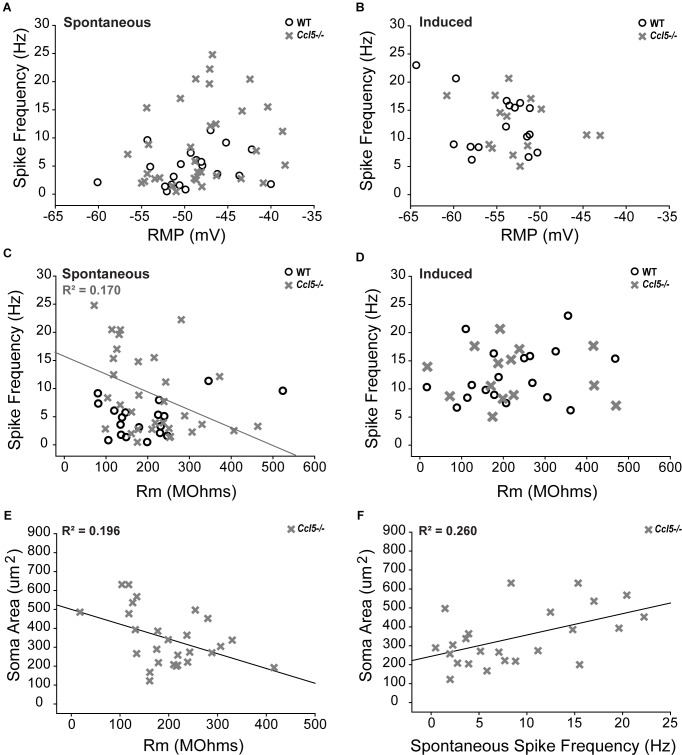
Increased spontaneous activity in *Ccl5^-/-^* RGCs is related to Rm and soma size. **(A,B)** Graphical representation of spontaneous **(A)** and induced **(B)** spiking frequency (Hz; *y*-axis) as a function of RMP (mV; *x*-axis) in RGCs from WT (white circles) and *Ccl5^-/-^* (gray crosses) mice reveals no relationship between these intrinsic properties in either condition for both genotypes (*p* > 0.05). **(C,D)** Graphical representation of spontaneous **(C)** and induced **(D)** spiking frequency (Hz; *y*-axis) as a function of Rm (MΩ; *x*-axis) in RGCs from WT (white circles) and *Ccl5^-/-^* (gray crosses) mice reveals no relationship between these intrinsic properties in either condition for WT RGCs (*p* > 0.05). Similarly, there is no relationship between Rm and induced spiking frequency in *Ccl5^-/-^* RGCs (*p* < 0.05). However, spontaneous spiking frequency is inversely proportional to Rm in *Ccl5^-/-^* RGCs (gray line; *R*^2^ = 0.170, *p* < 0.05). **(E)** Graphical representation of soma area (μm^2^; *y*-axis) as a function of Rm (MΩ; *x*-axis) in RGCs from *Ccl5^-/-^* (gray crosses) mice reveals that Rm is inversely proportional to soma area in *Ccl5^-/-^* RGCs (gray line; *R*^2^ = 0.196, *p* < 0.05). **(F)** Graphical representation of soma area (μm^2^; *y*-axis) as a function of spontaneous spiking frequency (Hz; *x*-axis) in RGCs from *Ccl5^-/-^* (gray crosses) mice reveals that spontaneous spiking frequency proportional to soma area in *Ccl5^-/-^* RGCs (gray line; *R*^2^ = 0.260, *p* < 0.05).

To better elucidate relationships between spike frequency and the physiological properties of RGCs in *Ccl5^-/-^* mice, we plotted spike frequency as function of both RMP and Rm in spontaneous and induced conditions. Based on Pearson Product Moment Correlation, there was no statistical relationship between spike frequency and RMP in either spontaneous or induced conditions (*p* > 0.05 for both; *n* = 20–33/group; **Figures 9A,B**). In contrast, spontaneous spike frequency negatively correlated with Rm in *Ccl5^-/-^* retina, such that RGCs with lower Rm exhibited higher spontaneous spiking frequencies (*p* < 0.05; *n* = 32/group; **Figure 9C**). Linear regression analysis revealed a low R-value for this relationship (*R*^2^ = 0.17; *p* < 0.01; *n* = 32/group), indicating that the relationship is not direct. Unlike *Ccl5^-/-^* RGCs, spontaneous spike frequency in WT RGCs did not correlate with Rm (*p* > 0.05; *n* = 19/group; **Figure 9C**). Similarly, there was no relationship between induced spike frequency and Rm in either *Ccl5^-/-^* or WT retina (*p* > 0.05 for both; *n* = 14–18/group; **Figure 9D**). Together, these data indicate the Ccl5-deficiency leads to increased spontaneous activity that is related to membrane resistance. Furthermore, this excitability phenotype can be abolished by direct application of current, which circumvents pre-synaptic circuitry.

Since pre-synaptic circuitry may contribute to hyperexcitability of RGCs in *Ccl5^-/-^* retina and membrane resistance is dependent, in part, on cell morphology, we examined the relationship between soma area and the electrophysiological characteristics of RGCs from *Ccl5^-/-^* retina. We found that soma area negatively correlated with Rm, such that RGCs with larger soma had lower Rm (*p* < 0.05; *n* = 27–28/group; **Figure 9E**). Like the relationship between Rm and spontaneous spiking frequency (**Figure 9C**), linear regression analysis revealed a rather low, but statistically significant, *R*^2^ value of 0.196 (*p* < 0.05; *n* = 27–28/group; **Figure 9E**). In contrast, soma area positively correlated with spontaneous spiking frequency, such that RGCs with larger soma tended to have a higher level of baseline activity (*p* < 0.05; *n* = 27–28/group; **Figure 9F**). Linear regression analysis revealed a higher, but still modest, *R*^2^ value of 0.260 (*p* < 0.05; *n* = 27–28/group; **Figure 9F**). There was no correlation between soma area and RMP (*p* > 0.05; *n* = 27–28/group). Together, these data suggest that Ccl5-dependent changes in Rm and spontaneous spiking frequency are both related to morphological changes in RGCs.

## Discussion

Here, we identified a role for Ccl5 signaling in development of the murine retina. We found that Ccl5 deficiency in *Ccl5^-/-^* mice results in thinning of the inner retina, particularly the INL and IPL (**Figure 1**). Assessment of postnatal timepoints important for development of the INL (P7 and P10) revealed Ccl5-dependent alterations in the cell density pattern and timing of apoptotic pruning (**Figure 2**). Assessment of major cell classifications in the mature inner retina revealed Ccl5-dependent changes in: (1) rod bipolar cell phenotypes, including displacement of their terminals from the IPL into the GCL (**Figure 3**), (2) RGC dendritic organization (**Figure 5**), and (3) amacrine cell morphology in the IPL (**Figure 7**). Examination of the intrinsic electrophysiological properties of RGCs revealed higher spontaneous activity in *Ccl5^-/-^* mice that was characterized by higher spiking frequency and a more depolarized resting potential (**Figure 8**). This hyperactive phenotype could be negated by current clamp, suggesting dependence on pre-synaptic circuitry (**Figure 8**). The putative relationship between Ccl5-dependent changes in pre-synaptic circuitry and RGC hyperactivity was further supported by statistical correlations between Rm, soma area and spontaneous spiking frequency (**Figure 9**). Together, our findings have implications for not only the functional role of Ccl5 in the developing retina, but also for the development of specific visual circuits within the retina more generally.

Despite CCL5-dependent changes in inner retinal development (**Figure 2**), our analyses in mature retina suggest that phenotypic changes in *Ccl5^-/-^* retina arise primarily from disorganized or aberrant wiring within the inner retina rather than loss of neurons. This is consistent with the magnitude of INL and IPL thinning, which was significant and highly consistent, but in the range of only 4–7% (**Figure 1**). We found evidence of aberrant organization of processes for all three of the major neuronal subtypes in the inner retina, including: bipolar cells, amacrine cells and RGCs (**Figures 3**, **5**, **7**). As a chemotactic cytokine, CCL5 induces migration of cells by influencing cell polarization, cytoskeleton dynamics and extracellular matrix adhesion (Weiss-Haljiti et al., 2004). In multiple contexts, CCL5-dependent modulation of these activities is linked to downstream activation and expression of a variety of cytoskeletal mediators, including the Rho family of GTPases, cyclin D1 and matrix metalloproteinases (Turner et al., 1995; Xia et al., 1996; Weiss-Haljiti et al., 2004; Murooka et al., 2008). Thus, it is conceivable that activation of Ccl5 signaling plays a role in physically attracting dendrites and terminals of synaptic partners together during wiring of inner retinal circuitry.

Our previous work indicates that Ccr5 and Ccr3, two high-affinity receptors for Ccl5, are expressed liberally in the inner retina (Duncan et al., 2017). The pattern of localization for Ccr5 and Ccr3 is similar to that of Ccl5 (Duncan et al., 2017), suggesting that Ccl5 could serve as a signaling factor between multiple cell types in the inner retina. Our current study is unable to determine the directional nature of Ccl5 signaling and thus, the cell type-specific dependency of Ccl5-mediated wiring. However, there is evidence that other chemokine signaling, i.e., Cxcl12, mediates tangential migration of interneurons during cortical development (Stumm et al., 2003, 2007; Daniel et al., 2005; Tiveron et al., 2006; Li et al., 2008; López-Bendito et al., 2008; Saìnchez-Alcañiz et al., 2011). Additionally, amacrine cells are early-born neurons, while bipolar cells are late-born neurons (Young, 1985; Ohsawa and Kageyama, 2008; Agathocleous and Harris, 2009; Mattar and Cayouette, 2015). Thus, bipolar cell orientation is dependent, in part, on proper positioning of the amacrine cell population (Reese and Keeley, 2016). Finally, our electrophysiological findings suggest that RGCs are hyperactive in *Ccl5^-/-^* mice (**Figure 8**), suggesting disruption of inhibitory neurotransmission. This is further supported by our findings that: (1) activity of *Ccl5^-/-^* and WT RGCs is indistinguishable when pre-synaptic circuitry is circumvented with current clamp (**Figure 8**) and (2) spontaneous spiking frequency correlates with both Rm and soma area (**Figure 9**). As such, amacrine cells may be preferentially impacted by Ccl5 signaling during inner retina development. Further studies are needed to determine the relevance of Ccl5 signaling for amacrine cell populations, which are the most diverse cell population in the retina in terms of both synaptic wiring and neurotransmission capabilities (Balasubramanian and Gan, 2014).

While the inner retina broadly exhibited changes in cytoarchitecture, the rod bipolar cell population was markedly affected with respect to both morphology and phenotype. This is significant from both CCL5 and retinal circuitry perspectives. Ccl5-mediated activity, including cell migration and angiogenic outcomes, in non-neuronal cell types is PKCα dependent (Maillard et al., 2014; Ya et al., 2018). We found that Ccl5 deficiency markedly reduces GFP expression under the GNAT3 promoter, which encodes expression of GUS. GUS localizes to rod bipolar cell terminals in mammalian retina (Son et al., 2011). This is interesting in light of our finding that rod bipolar cells in *Ccl5^-/-^* retina terminate improperly beyond the boundaries of the IPL (**Figure 3**). It is possible that GNAT3 expression in rod bipolar cells is either dependent upon or mediates establishment of rod bipolar cell terminals. Although we did not observe Ccl5-dependent alterations in the morphology of Type 7 cone bipolar cells in Gus^gfp^ and Gus^gfp^/*Ccl5^-/-^* mice, the potential remains that Ccl5 signaling could play a role in the development, differentiation or synaptic partnering of other cone bipolar cells. This should be systematically examined in future studies.

Here, we focused on developmental outcomes of Ccl5 signaling. However, Ccl5 machinery is expressed constitutively in mature mouse retina (Duncan et al., 2017). This suggests that Ccl5 signaling is likely important for inner retinal neurons during both development and maturity. In the mature CNS, Ccl5 signaling linked to both inhibitory and glutamatergic neurotransmission. Ccl5 and Ccr expression is present within the ventral tegmental area, nucleus accumbens, striatum, prefrontal cortex, hippocampus, striatum and frontal cortex (Szabo et al., 2002; Campbell et al., 2013; Mocchetti et al., 2013; Fe Lanfranco et al., 2018). Given expression of Ccl5 and Ccrs are associated with known brain circuits, i.e., dopaminergic mesolimbic pathway, and can modulate neurotransmission, it has been proposed that chemokines may serve as a third neurotransmitter system in the CNS (Adler and Rogers, 2005). Thus, it is possible that Ccl5 signaling is preserved in mature retina as a modulator of neurotransmission between bipolar cells, amacrine cells and RGCs. This would have additional implications for our findings regarding RGC hyperactivity in *Ccl5^-/-^* mice (**Figure 8**).

Overall, our findings identify Ccl5 signaling as a mediator of inner retinal circuitry during development of the murine retina. Additional studies are needed to determine the specific cell-cell interactions that mediate this signaling as well as the temporal characteristics during development and in maturity. The apparent role of Ccl5 in retinal development further supports chemokines as trophic modulators of CNS development and function that extends far beyond the inflammatory contexts in which they were first characterized.

## Author Contributions

DD and RW equally designed the study, performed the experiments, analyzed the data, and contributed to writing of themanuscript. AR, ES, CW, MR and CF performed the experiments and analyzed the data. All authors have read and approved the final manuscript.

## Conflict of Interest Statement

The authors declare that the research was conducted in the absence of any commercial or financial relationships that could be construed as a potential conflict of interest.

## Supplementary Material

FIGURE S1 Ccl5-dependent increases in mean spontaneous spiking frequency is consistent across RGC sub-types. Graphical representation of mean spontaneous spiking frequency (Hz; *y*-axis) in ON or OFF RGCs (left; *x*-axis) and ON/OFF RGCs (right; *x*-axis) in WT (white) and *Ccl5^-/-^* (gray) retina. Spontaneous spiking frequency is 39% higher in ON or OFF RGCs (*n* = 7) and 45% higher in ON/OFF RGCs (*n* = 11) in *Ccl5^-/-^* mice, as compared to WT retina (*n* = 8 for both; *p* < 0.05). Error bars indicate standard deviation. Asterisks indicate *p* < 0.05.

## Abbreviations

Ccl5: C-C motif chemokine ligand 5
CNS: central nervous system
CTB: cholera toxin beta-subunit
GCL: ganglion cell layer
GFP: green fluorescent protein
GNAT3: guanine nucleotide binding protein, alpha transducing 3
GUS: gustducin
INL: inner nuclear layer
IPL: inner plexiform layer
NFL: nerve fiber layer
OD: optic disk
ONH: optic nerve head
ONL: outer nuclear layer
OPL: outer plexiform layer
P7: postnatal day 7
P10: postnatal day 10
PKCα: protein kinase c alpha
RANTES: regulated on activation, normal T cell expressed and secreted
RGC: retinal ganglion cell
SC: superior colliculus
SD-OCT: spectral domain optical coherence tomography

## References

[B1] Adler M. W. Rogers T. J. (2005). Are chemokines the third major system in the brain? *J. Leukoc. Biol.* 78 1204–1209. 10.1189/jlb.0405222 16204637

[B2] Agathocleous M. Harris W. A. (2009). From progenitors to differentiated cells in the vertebrate retina. *Ann. Rev. Cell Dev. Biol.* 25 45–69. 10.1146/annurev.cellbio.042308.113259 19575661

[B3] Appay V. Rowland-Jones S. L. (2001). RANTES: a versatile and controversial chemokine. *Trends Immunol.* 22 83–87. 10.1016/S1471-4906(00)01812-3 11286708

[B4] Babcock A. A. Kuziel W. A. Rivest S. Owens T. (2003). Chemokine expression by glial cells directs leukocytes to sites of axonal injury in the CNS. *J. Neurosci.* 23 7922–7930. 10.1523/JNEUROSCI.23-21-07922.2003 12944523 PMC6740601

[B5] Balasubramanian R. Gan L. (2014). Development of retinal amacrine cells and their dendritic stratification. *Curr. Ophthalmol. Rep.* 2 100–106. 10.1007/s40135-014-0048-2 25170430 PMC4142557

[B6] Bond W. S. Hines-Beard J. B. GoldenMerry Y. P. L. Davis M. Farooque A. Sappington R. M. (2016). Virus-mediated EpoR76E therapy slows optic nerve axonopathy in experimental glaucoma. *Mol. Ther.* 2 230–239. 10.1038/mt.2015.198 26502777 PMC4817814

[B7] Brandstätter J. H. Koulen P. Wässle H. (1998). Diversity of glutamate receptors in the mammalian retina. *Vision Res.* 38 1385–1397. 10.1016/S0042-6989(97)00176-4 9667006

[B8] Campbell L. A. Avdoshina V. Rozzi S. Mocchetti I. (2013). CCL5 and cytokine expression in the rat brain: differential modulation by chronic morphine and morphine withdrawal. *Brain Behav. Immun.* 34 130–140. 10.1016/j.bbi.2013.08.006 23968971 PMC3795805

[B9] Crish S. D. Sappington R. M. Inman D. M. Horner P. J. Calkins D. J. (2010). Distal axonopathy with structural persistence in glaucomatous neurodegeneration. *Proc. Nat. Acad. Sci. U.S.A.* 107 5196–5201. 10.1073/pnas.0913141107 20194762 PMC2841892

[B10] Daniel D. Rossel M. Seki T. König N. (2005). Stromal cell-derived factor-1 (SDF-1) expression in embryonic mouse cerebral cortex starts in the intermediate zone close to the pallial-subpallial boundary and extends progressively towards the cortical hem. *Gene Expr. Patterns* 5 317–322. 10.1016/j.modgep.2004.10.007 15661637

[B11] Duncan D. S. McLaughlin W. M. Vasilakes N. Echevarria F. D. Formichella C. R. Sappington R. M. (2017). Characterization of constitutive and stress-induced Ccl5 signaling in rodent retina. *J. Clin. Cell. Immunol.* 8:506. 10.4172/2155-9899.1000506 28936366 PMC5604884

[B12] Echevarria F. D. Formichella C. R. Sappington R. M. (2017). Interleukin-6 deficiency attenuates retinal ganglion cell axonopathy and glaucoma-related vision loss. *Front. Neurosci.* 11:318. 10.3389/fnins.2017.00318 28620279 PMC5450377

[B13] Echevarria F. D. Walker C. C. Abella S. K. Won M. Sappington R. M. (2013). Stressor-dependent alterations in glycoprotein 130: implications for glial cell reactivity, cytokine signaling and ganglion cell health in glaucoma. *J. Clin. Exp. Ophthalmol.* 4:1000286. 25018894 10.4172/2155-9570.1000286 PMC4091850

[B14] Fe Lanfranco M. Mocchetti I. Burns M. P. Villapol S. (2018). Glial- and neuronal-specific expression of CCL5 mRNA in the rat brain. *Front. Neuroanat.* 11:137. 10.3389/fnana.2017.00137 29375328 PMC5770405

[B15] Galasso J. M. Harrison J. K. Silverstein F. S. (1998). Excitotoxic brain injury stimulates expression of the chemokine receptor CCR5 in neonatal rats. *Am. J. Pathol.* 153 1631–1640. 10.1016/S0002-9440(10)65752-5 9811356 PMC1853404

[B16] Gamo K. Kiryu-Seo S. Konishi H. Aoki S. Matsushima K. Wada K. (2008). G-protein-coupled receptor screen reveals a role for chemokine receptor CCR5 in suppressing microglial neurotoxicity. *J. Neurosci.* 28 11980–11988. 10.1523/JNEUROSCI.2920-08.2008 19005063 PMC6671655

[B17] Ghosh K. K. Bujan S. Haverkamp S. Feigenspan A. Wässle H. (2004). Types of bipolar cells in the mouse retina. *J. Comp. Neurol.* 469 70–82. 10.1002/cne.10985 14689473

[B18] He J. Chen Y. Farzan M. Choe H. Ohagen A. Gartner S. (1997). CCR3 and CCR5 are co-receptors for HIV-1 infection of microglia. *Nature* 385 645–649. 10.1038/385645a0 9024664

[B19] Huang L. Max M. Margolskee R. F. Su H. Masland R. H. Euler T. (2003). Gprotein subunit G gamma 13 is coexpressed with G alpha o, G beta 3, and G beta 4 in retinal ON bipolar cells. *J. Comp. Neurol.* 455 1–10. 10.1002/cne.10396 12454992

[B20] Jeon C.-J. Strettoi E. Masland R. H. (1998). The major cell populations of the mouse retina. *J. Neurosci.* 18 8936–8946. 10.1523/JNEUROSCI.18-21-08936.1998 9786999 PMC6793518

[B21] Li G. Adesnik H. Li J. Long J. Nicoll R. A. Rubenstein J. L. R. (2008). Regional distribution of cortical interneurons and development of inhibitory tone are regulated by Cxcl12/Cxcr4 signaling. *J. Neurosci.* 28 1085–1098. 10.1523/JNEUROSCI.4602-07.2008 18234887 PMC3072297

[B22] López-Bendito G. Sánchez-Alcañiz J. A. Pla R. Borrell V. Picoì E. Valdeolmillos M. (2008). Chemokine signaling controls intracortical migration and final distribution of GABAergic interneurons. *J. Neurosci.* 28 1613–1624. 10.1523/JNEUROSCI.4651-07.2008 18272682 PMC6671533

[B23] Maillard L. Saito N. Hlawaty H. Friand V. Suffee N. Chmilewsky F. (2014). RANTES/CCL5 mediated-biological effects depend on the syndecan-4/PKCa signaling pathway. *Biol. Open* 3 995–1004. 10.1242/bio.20148227 25260916 PMC4197448

[B24] Mattar P. Cayouette M. (2015). Mechanisms of temporal identity regulation in mouse retinal progenitor cells. *Neurogenesis* 2:e1125409. 10.1080/23262133.2015.1125409 27606333 PMC4973599

[B25] Mennicken F. Chabot J. G. Quirion R. (2002). Systemic administration of kainic acid in adult rat stimulates expression of the chemokine receptor CCR5 in the forebrain. *Glia* 37 124–138. 10.1002/glia.10021 11754211

[B26] Mocchetti I. Campbell L. A. Harry G. J. Avdoshina V. (2013). When human immunodeficiency virus meets chemokines and microglia: neuroprotection or neurodegeneration? *J. Neuroimmune Pharmacol.* 8 118–131. 10.1007/s11481-012-9353-4 22527632 PMC3427402

[B27] Morgan J. Wong R. (2005). “Development of cell types and synaptic connections in the retina,” in *Webvision: The Organization of the Retina and Visual System*, eds Kolb H. Fernandez E. Nelson R. (Salt Lake City: UT: University of Utah Health Sciences Center). 21413410

[B28] Murooka T. T. Rahbar R. Platanias L. C. Fish E. N. (2008). CCL5-mediated T-cell chemotaxis involves the initiation of mRNA translation through mTOR/4E-BP1. *Blood* 111 4892–4901. 10.1182/blood-2007-11-125039 18337562 PMC2384123

[B29] Ohsawa R. Kageyama R. (2008). Regulation of retinal cell fate specification by multiple transcription factors. *Brain Res.* 1192 90–98. 10.1016/j.brainres.2007.04.014 17488643

[B30] Pham V. T. Wen L. McCluskey P. Madigan M. C. Penfold P. L. (2005). Human retinal microglia express candidate receptors for HIV-1 infection. *Br. J. Ophthalmol.* 89 753–757. 10.1136/bjo.2004.057828 15923514 PMC1772690

[B31] Reese B. E. (2011). Development of the retina and optic pathway. *Vision Res.* 51 613–632. 10.1016/j.visres.2010.07.010 20647017 PMC2974959

[B32] Reese B. E. Keeley P. W. (2016). Genomic control of neuronal demographics in the retina. *Prog. Retin. Eye Res.* 55 246–259. 10.1016/j.preteyeres.2016.07.003 27492954 PMC5112127

[B33] Rossi D. Zlotnik A. (2000). The biology of chemokines, and their receptors. *Annu. Rev. Immunol.* 18 217–242. 10.1146/annurev.immunol.18.1.217 10837058

[B34] Sappington R. M. Carlson B. J. Crish S. Calkins D. J. (2010). The microbead occlusion model: a paradigm for induced ocular hypertension in rats, and mice. *Invest. Ophthalmol. Vis. Sci.* 51 207–216. 10.1167/iovs.09-3947 19850836 PMC2869054

[B35] Saìnchez-Alcañiz J. A. Haege S. Mueller W. Pla R. Mackay F. Schulz S. (2011). Cxcr7 controls neuronal migration by regulating chemokine responsiveness. *Neuron* 69 77–90. 10.1016/j.neuron.2010.12.006 21220100

[B36] Sims S. M. Holmgren L. Cathcart H. M. (2012). Sappington RM. Spatial regulation of interleukin-6 signaling in response to neurodegenerative stressors in the retina. *Am. J. Neurodegen. Dis.* 1 168–179. 23024928 PMC3560463

[B37] Son M. J. Hu Y. J. Kim S. A. Chun M. H. Kim I. B. Kim M. S. (2011). Expression of α-gustducin in mammalian retinas. *Neuroreport* 22 146–150. 10.1097/WNR.0b013e328343701f 21200350

[B38] Sorce S. Bonnefont J. Julien S. Marq-Lin N. Rodriguez I. Dubois-Dauphin M. (2010). Increased brain damage after ischaemic stroke in mice lacking the chemokine receptor CCR5. *Br. J. Pharmacol.* 160 311–321. 10.1111/j.1476-5381.2010.00697.x 20423342 PMC2874853

[B39] Sorce S. Myburgh R. Krause K. H. (2011). The chemokine receptor CCR5 in the central nervous system. *Prog. Neurobiol.* 93 297–311. 10.1016/j.pneurobio.2010.12.003 21163326

[B40] Stumm R. Kolodziej A. Schulz S. Kohtz J. D. Höllt V. (2007). Patterns of SDF-1alpha and SDF-1gamma mRNAs, migration pathways, and phenotypes of CXCR4-expressing neurons in the developing rat telencephalon. *J. Comp. Neurol.* 502 382–399. 10.1002/cne.21336 17366607

[B41] Stumm R. K. Zhou C. Ara T. Lazarini F. Dubois-Dalcq M. Nagasawa T. (2003). CXCR4 regulates interneuron migration in the developing neocortex. *J. Neurosci.* 23 5123–5130. 10.1523/JNEUROSCI.23-12-05123.2003 12832536 PMC6741192

[B42] Subileau E. A. Rezaie P. Davies H. A. Colyer F. M. Greenwood J. Male D. K. (2009). Expression of chemokines and their receptors by human brain endothelium: implications for multiple sclerosis. *J. Neuropathol. Exp. Neurol.* 68 227–240. 10.1097/NEN.0b013e318197eca7 19225413

[B43] Szabo I. Chen X. H. Xin L. Adler M. W. Howard O. M. Oppenheim J. J. (2002). Heterologous desensitization of opioid receptors by chemokines inhibits chemotaxis and enhances the perception of pain. *Proc. Natl. Acad. Sci. U.S.A.* 2002 10276–10281. 10.1073/pnas.102327699 12130663 PMC124904

[B44] Tiveron M.-C. Rossel M. Moepps B. Zhang Y. L. Seidenfaden R. Favor J. (2006). Molecular interaction between projection neuron precursors and invading interneurons via stromal-derived factor 1 (CXCL12)/CXCR4 signaling in the cortical subventricular zone/intermediate zone. *J. Neurosci.* 26 13273–13278. 10.1523/JNEUROSCI.4162-06.2006 17182777 PMC6674999

[B45] Tripathy D. Thirumangalakudi L. Grammas P. (2010). RANTES upregulation in the Alzheimer’s disease brain: a possible neuroprotective role. *Neurobiol. Aging* 31 8–16. 10.1016/j.neurobiolaging.2008.03.009 18440671 PMC2803489

[B46] Turner L. Ward S. G. Westwick J. (1995). RANTES-activated human T lymphocytes. A role for phosphoinositide 3-kinase. *J. Immunol.* 155 2437–2444. 7544376

[B47] Weiss-Haljiti C. Pasquali C. Ji H. Gillieron C. Chabert C. Curchod M. L. (2004). Involvement of phosphoinositide 3-kinase gamma, rac, and PAK signaling in chemokine-induced macrophage migration. *J. Biol. Chem.* 279 43273–43284. 10.1074/jbc.M402924200 15292195

[B48] Weitlauf C. Ward N. Lambert W. Sidorova T. Ho K. Sappington R. M. (2015). Transiently increased TRPV1 mediates stress-induced enhancement of neuronal excitation. *J. Neurosci.* 34 15369–15381. 10.1523/JNEUROSCI.3424-14.2014 PMC4228139 25392504

[B49] Xia M. Gaufo G. O. Wang Q. Sreedharan S. P. Goetzl E. J. (1996). Transduction of specific inhibition of HuT 78 human T cell chemotaxis by type I vasoactive intestinal peptide receptors. *J. Immunol.* 157 1132–1138. 8757618

[B50] Ya F. Zhang P. Zeshong T. Li Q. Ling W. Yang Y. (2018). Coenzyme Q10 reduces platelet hyperreactivity and attenuates atherosclerosis via inhibiting platelet αIIbβ3-mediated signaling pathway. *Atherosclerosis* 32 126–127. 10.1016/j.atherosclerosissup.2018.04.389

[B51] Young R. W. (1985). Cell differentiation in the retina of the mouse. *Anat. Rec.* 212 199–205. 10.1002/ar.1092120215 3842042

[B52] Zhang X. Serb J. M. Greenlee M. H. W. (2011). Mouse retinal development: a dark horse model for systems biology research. *Bioinform. Biol. Insights* 5 99–113. 10.4137/BBI.S6930 21698072 PMC3118678

