# Supplementary figures and images for: Ccl5 Mediates Proper Wiring of Feedforward and Lateral Inhibition Pathways in the Inner Retina
Source: Front Neurosci. 2018 Oct 12;12:702. doi: 10.3389/fnins.2018.00702 (PMC6194164; doi:10.3389/fnins.2018.00702)

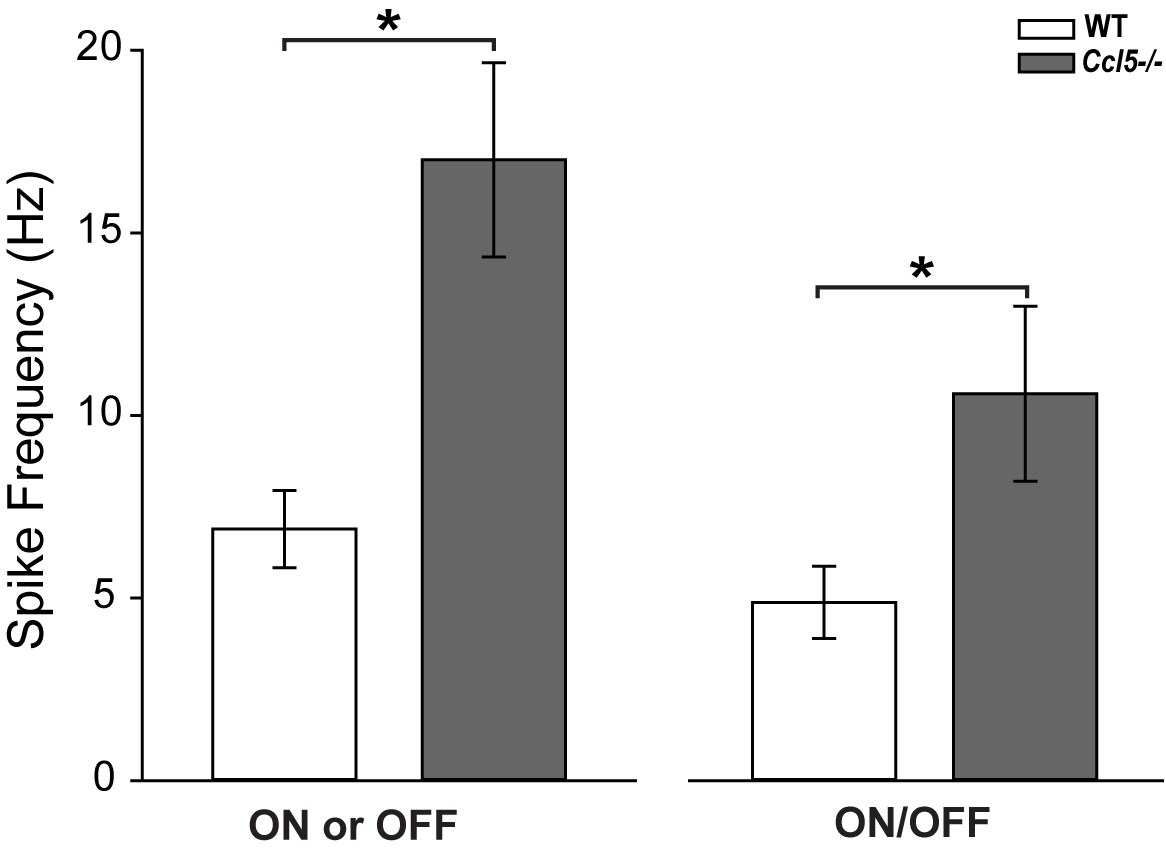

Supplement: FIGURE S1 — Ccl5-dependent increases in mean spontaneous spiking frequency is consistent across RGC sub-types. Graphical representation of mean spontaneous spiking frequency (Hz; y-axis) in ON or OFF RGCs (left; x-axis) and ON/OFF RGCs (right; x-axis) in WT (white) and Ccl5-/- (gray) retina. Spontaneous spiking frequency is 39% higher in ON or OFF RGCs (n = 7) and 45% higher in ON/OFF RGCs (n = 11) in Ccl5-/- mice, as compared to WT retina (n = 8 for both; p < 0.05). Error bars indicate standard deviation. Asterisks indicate p < 0.05. [file Image_1.TIF]
